# Outcomes associated with scale-up of the Stepping On falls prevention program: A case study in redesigning for dissemination

**DOI:** 10.1017/cts.2020.17

**Published:** 2020-03-04

**Authors:** Jane E. Mahoney, Ron Gangnon, Lindy Clemson, LaVerne Jaros, Sandy Cech, Jill Renken

**Affiliations:** 1 Department of Medicine, University of Wisconsin School of Medicine and Public Health, Madison, WI, USA; 2 Department of Biostatistics and Medical Informatics, University of Wisconsin School of Medicine and Public Health, Madison, WI, USA; 3 Faculty of Medicine and Health, University of Sydney, Sydney, Australia; 4 Kenosha County Aging and Disability Resource Center, Kenosha, WI, USA; 5 Captain James A. Lovell Veterans Administration Hospital, North Chicago, IL, USA; 6 Wisconsin Institute for Healthy Aging, Madison, WI, USA

**Keywords:** Implementation, fidelity, falls prevention, evidence-based programs, dissemination

## Abstract

**Introduction:**

Translating complex behavior change interventions into practice can be accompanied by a loss of fidelity and effectiveness. We present the evaluation of two sequential phases of implementation of a complex evidence-based community workshop to reduce falls, using the Replicating Effective Programs Framework. Between the two phases, workshop training and delivery were revised to improve fidelity with key elements.

**Methods:**

Stepping On program participants completed a questionnaire at baseline (phase 1: *n* = 361; phase 2: *n* = 2219) and 6 months post-workshop (phase 1: *n* = 232; phase 2: *n* = 1281). Phase 2 participants had an additional follow-up at 12 months (*n* = 883). Outcomes were the number of falls in the prior 6 months and the Falls Behavioral Scale (FaB) score.

**Results:**

Workshop participation in phase 1 was associated with a 6% reduction in falls (RR = 0.94, 95% CI 0.74–1.20) and a 0.14 improvement in FaB score (95% CI, 0.11– 0.18) at 6 months. Workshop participation in phase 2 was associated with a 38% reduction in falls (RR = 0.62, 95% CI 0.57–0.68) and a 0.16 improvement in FaB score (95% CI 0.14–0.18) at 6 months, and a 28% reduction in falls (RR = 0.72, 95% CI 0.65–0.80) and a 0.19 score improvement in FaB score (95% CI 0.17–0.21) at 12-month follow-up.

**Conclusions:**

Effectiveness can be maintained with widespread dissemination of a complex behavior change intervention if attention is paid to fidelity of key elements. An essential role for implementation science is to ensure effectiveness as programs transition from research to practice.

## Introduction

In the United States, there is little data about the effectiveness of evidence-based falls prevention programs when implemented in community settings. Following adoption, the next phase of implementation should be concerned with strategies for improving program fidelity in the field [1]. Given the potential “voltage drop” in effectiveness that occurs with dissemination [2], it is important to conduct program evaluation to assure continued effectiveness. We present the findings of two sequential pre–post evaluations that affirm the importance of attention to key elements of intervention delivery.

Stepping On is a community-based falls prevention program that was tested in Australia. Originally led by occupational therapists (OTs), it was shown to reduce falls by 31% in a randomized controlled trial [3]. We (co-author Mahoney and the Kenosha County Aging and Disability Resource Center) brought it to Wisconsin in 2006. We were concerned that county Aging Units would not have access to OTs to lead the workshop, so we permitted Aging Units to use other professionals, including non-health professionals, to lead the workshops. Aging Unit leaders were enthusiastic about adopting, implementing, and sustaining the program; they even began to train individuals in neighboring counties to implement the program. However, adaptation and implementation were done without attention to fidelity; training was minimal; and each county made their own modifications without attention to key elements. Indeed, there had been no elucidation of key elements, and it was unclear which components of the program were essential and which could be changed or omitted without loss of fidelity. Early evaluation of outcomes suggested fidelity may have been compromised; there was no reduction in falls in association with the program.

The Centers for Disease Control and Prevention provided funding in 2007 for a dissemination and implementation research study, which would identify the key elements of Stepping On and build a program package to ensure fidelity of delivery and effectiveness, and maximize dissemination, implementation, and sustainment of the program nationally. Through the CDC award, from 2007 to 2012, we used the Replicating Effective Programs (REP) framework [4] to research and improve Stepping On program implementation. Simultaneous with the work of the CDC grant, which was conducted in three sites in Wisconsin, we were disseminating Stepping On more widely across Wisconsin through funding from the Administration on Aging (now Administration for Community Living). Findings from the CDC grant immediately and continuously informed the Administration for Community Living-funded dissemination of Stepping On. Revisions based on the CDC study, including training on key elements, were provided in real time to existing leaders and integrated into trainings for new leaders. From 2008 to 2011, we evaluated the outcomes associated with implementing Stepping On in Wisconsin using the same methodology and tools that were used for the initial evaluation. This afforded us a unique natural experiment to see if outcomes would improve as fidelity was improved.

This paper presents pre–post evaluation findings from the two phases of implementing Stepping On. The first implementation phase, 2006 to January 2009, encompassed the initial adoption into eight counties. The second phase, 2008–2011, which encompassed the broader dissemination in Wisconsin, coincided with the changes in the Stepping On implementation package to improve fidelity. Our primary objective was to determine if implementation of the revised Stepping On training and implementation package, with the enhancements to improve fidelity, would be associated with a reduction in the rate of falls among participants. We secondarily sought to determine if a reduction in the rate of falls would differ by location of workshop (rural versus urban) or by type of leader providing the workshop.

## Methods

### Stepping On Fall Prevention Program

Stepping On is a small-group, self-efficacy-based workshop addressing multiple components of fall risk over seven weekly sessions and a 3-month booster. Group size is typically 10–14 participants. A trained health professional facilitates the sessions, and guest experts (physical therapist (PT), OT, pharmacist, low vision expert, community safety expert) attend selected sessions to address fall risk factors, including low vision, home safety hazards, medications, and risky behaviors such as walking unsafely. A PT attends session 1 to teach balance and strength exercises, which are then practiced daily at home and progressed throughout the sessions. In the original study, community-dwelling adults aged ≥70 who had a fall in the past year or a fear of falling, who were able to walk at least 10 feet without the aid of another person (but could use a cane or a walker), and who were cognitively intact were eligible to participate in the program [5].

### Phase 1 Implementation of Stepping On (July 2006–January 2009)

#### Modifications to Stepping On

When starting Stepping On in Wisconsin in 2006, we modified the Australian program to increase feasibility of adoption and implementation by deleting the home visit, which in the original program occurred 2 weeks after the final session, and broadening the criteria for who could enroll in the workshop to also include older adults younger than age 70 who were community-dwelling, cognitively intact, and able to walk at least 10 feet without the aid of another person. We also broadened the criteria for a Stepping On leader. The original Stepping On manual stated “This manual is for occupational therapists, physiotherapists, and other health professionals and health promotion workers in the area of falls-prevention with older people” [5]. We included people who lacked health degrees but who worked with older adults in a professional capacity. Of the nine leaders trained in phase 1, two were registered nurses (RNs); the others were health educators (2), directors of Aging Units (2), a social worker, a rehabilitation counselor, and a registered dietician. In an attempt to mitigate potential loss of effectiveness from deletion of the home visit and the change in prerequisites for Stepping On leaders, we added two additional booster sessions for a total of three boosters over 6 months, rather than the original one booster at 3 months post-program. We also added a lay co-leader, based on our positive experience with the Chronic Disease Self-Management Program [6]. Leaders provided feedback on their first Stepping On workshops, which was incorporated into a second North American edition of Stepping On [7]. Changes in the second North American edition, compared to the original Australian edition, consisted primarily of reformatting of session materials to provide more explicit instructions to leaders and to improve usability. We created the first draft of a leader training manual in 2007.

#### Implementation and evaluation

Phase 1 Stepping On was implemented in eight counties from July 2006 to January 2009 and evaluated through funding from the Wisconsin Partnership Program. In May 2006, nine professionals were trained as leaders, and three older adults were trained as lay leaders through a 2-day training. The trainees read the Australian leader manual [5], which provided program content along with background information on conceptual underpinnings of the program. Phone consultations with Dr. Clemson, the program originator, addressed questions. In general, the trainees focused on understanding the program process and learning how to run the program and administer the evaluation tool. The project coordinator (who was also a trainee) was an RN who was experienced in multifactorial falls assessment [8] and was a Master Trainer for the Chronic Disease Self-Management Program [6]. She added to the training by contributing information on falls risk factors and falls prevention, and addressing marketing and recruitment, based on her previous experiences. The project coordinator trained 19 new leaders and peer leaders in the eight counties through additional 2½-day trainings in May and December 2007. Training was formalized into a training manual and trainee certification process, but content was similar to the first training.

For the phase 1 evaluation, the Stepping On leader invited all workshop participants to participate in a program evaluation, consisting of a baseline questionnaire before session 1 and a follow-up questionnaire 6 months after the last session. Our program evaluation received an exemption from the University of Wisconsin Institutional Review Board.

### Phase 2 Implementation of Stepping On (2008–2011): Using the REP Framework to Improve Implementation

#### REP framework

**Fig. 1.**
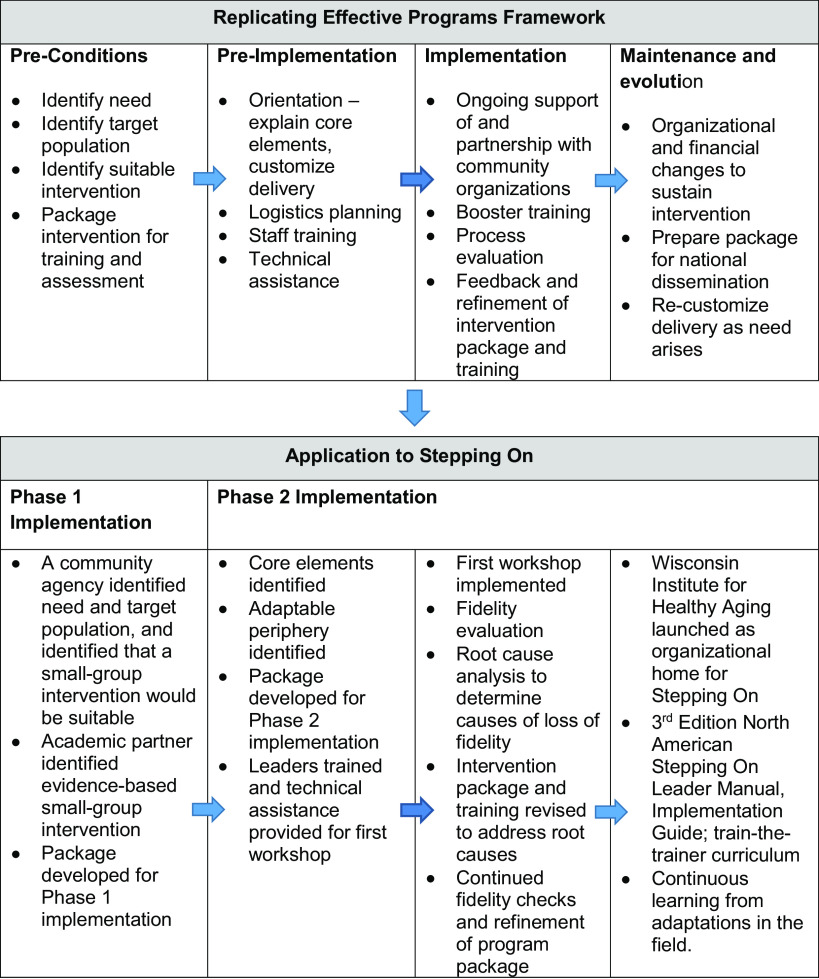
Application of Replicating Effective Programs framework to Stepping On.

The REP framework was developed by the Centers for Disease Control and Prevention as a blueprint to assist with the systematic dissemination and implementation of behavioral and treatment interventions in community settings, in such a way that fidelity is maintained while adaptability is fostered, toward an ultimate goal of maximizing broad adoption, reach, effectiveness, and sustainment of healthcare interventions [4]. Figure 1 shows the four stages of the REP framework: pre-conditions, pre-implementation, implementation, and maintenance and evolution. The effectiveness of REP as a dissemination and implementation framework was demonstrated in a national randomized controlled trial examining its use to disseminate HIV treatment interventions in community settings [9,10].

#### Improving stepping on implementation

Through funding from the CDC, we accomplished the “pre-implementation” and “implementation” phases of the REP framework, as shown in Fig. 1. First, we elucidated key elements of Stepping On and identified areas to adapt based on interviews and focus groups with prior participants, leaders, and guest experts engaged in Stepping On (i.e., customized delivery). Second, we trained and provided technical assistance to a new Stepping On leader who implemented the workshop. We evaluated every session for fidelity and identified areas where fidelity was lost, then conducted a root cause analysis to establish root causes and solutions to losses of fidelity. Based on the solutions we identified, we reworked leader training, the leader program manual, and the criteria for who could be a leader (Table 1). We revised the criteria for who could enroll in the workshop to exclude individuals who required a walker for indoor walking. We developed an implementation guide to coach organizations on how to prepare for and implement the workshop. The result was a comprehensive program package to support high fidelity implementation [11–13].

**Table 1.** Revisions to Stepping On program package with phase 2 implementation

Year	Change
2008	Number of booster sessions reduced from 3 to 1 Return to the requirement of either an in-home visit or phone call within 2 weeks after session 7 Modifications to session content and format to increase usability and participant uptake of information Extension of training for new leaders to 3 days Institution of fidelity monitoring of a Stepping On session for each newly trained leader
2009–2010	Changes to manual and training to incorporate key elements from Delphi consensus and feedback from focus groups and interviews of leaders, invited experts, and participants Dissemination of key elements to all existing leaders Changes to eligibility requirements for leaders, now requiring them to be nurses, physical or occupational therapists or therapy assistants, social workers or health educators, based on the Delphi consensus Further revisions to the leader manual and supporting materials to enhance fidelity of program delivery, based on the findings from fidelity monitoring at each session of one workshop Further revisions to leader training, incorporating more brainstorming, practice of exercises, and role-playing with feedback, in order to improve leaders’ self-efficacy to lead and advance balance and strength exercises, practical skills related to program implementation, and knowledge and skills related to key elements of adult learning and group facilitation Institution of competency evaluation at the end of training, including an open-book quiz on knowledge of causes and prevention of falls, a closed-book quiz on key elements, and demonstration of ability to use adult learning and facilitation skills, and lead and advance exercises Changes to eligibility requirements for participants, requiring that they be able to ambulate in the home without the use of a walker. 1-day booster trainings to retrain all existing Stepping On leaders
2010–2011	Creation of DVDs of falls risk vignettes for use in Stepping On sessions Third North American leader manual and DVDs provided to all existing Stepping On leaders.

Simultaneous with the work of the CDC study, we disseminated Stepping On throughout Wisconsin through funding from the Administration for Community Living. Incorporating the findings generated from the CDC-sponsored study on Stepping On, we iteratively modified aspects of training and delivery of Stepping On in Wisconsin from 2008 to 2011, as shown in Table 1. In general, changes improved fidelity by enhancing leader knowledge and self-efficacy of key elements, and ensuring leader competency in group facilitation, adult learning principles, and how to lead and progress balance and strength exercises. The Stepping On lead trainer iteratively disseminated changes to all current Stepping On leaders. Any new trainings utilized new materials.

#### Phase 2 implementation and evaluation

The Wisconsin Department of Health and Family Services’ Divisions of Disability and Elder Services, through funding from the federal Administration for Community Living, led the implementation and evaluation of Stepping On across Wisconsin from January 2008 through June 2011.

Leaders were identified by Aging Units in each county. Leaders were volunteer or were paid by the organization that sponsored them (Aging and Disability Resource Center, Aging Unit, healthcare organization, etc.). Leaders worked with sponsoring organizations in their communities to recruit participants and guest experts and facilitate the workshop. Guest experts (PT, low vision expert, pharmacist, community safety officer) were not paid by the program. Participants completed a registration form that screened for falls, fear of falling, and use of a walker indoors. Workshop coordinators or leaders screened out individuals who were noted to have obvious cognitive impairment during the registration process. Some leaders provided the workshop free to participants. In other cases, there was a cost of up to $30.00 for participants.

Phase 2 evaluation occurred for workshops implemented between January 2008 and June 2011. Leaders were trained from October 2006 through September 2010. A 1-day refresher training was offered in 2009 to bring leaders trained prior to 2009 up-to-date. Leaders were encouraged to participate in monthly calls with the Stepping On master trainer, which afforded the opportunity to provide updates and reinforce key elements. All new leaders trained from 2008 received one fidelity check followed by a one-on-one coaching session with the master trainer.

For the phase 2 evaluation, similar to phase 1, Stepping On leaders invited participants to participate in a program evaluation, consisting of a baseline questionnaire before session 1 and a follow-up questionnaire at 6 and 12 months after the last session. All participants from all workshops held in Wisconsin during the evaluation period were included in the evaluation. The phase 2 program evaluation received an exemption from the University of Wisconsin Institutional Review Board.

### Data Collection

The baseline questionnaire in both phases was completed within 1 week before the first workshop session. It included demographic information (age, gender, race, ethnicity, marital status), use of an assistive device for indoor walking, number of falls in the prior 6 months, and occurrence of any injurious fall (i.e., requiring medical attention). Participants also completed a measure of fall behavioral risk. The number of falls was based on the question, “How many times have you fallen in the last 6 months?” Fall behavioral risk was determined using the Falls Behavioral Scale (FaB) [14]. This measure was selected as it had shown significance between treatment and control groups in the Stepping On randomized controlled trial. FaB evaluates behavioral factors affecting falls risk. Scores range from 1 (most risky) to 4 (most protective). The tool has good internal consistency (Cronbach α 0.84) and test–retest reliability (ICC ranging from 0.78 to 0.96 for factor subscales).

Attendance at weekly sessions was tracked by the leader and returned to the research assistant at the end of the workshop. Participants were deemed “completers” if they attended at least five of the seven sessions.

Participants were mailed a follow-up questionnaire at 6 months after the end of session 7 (the last session), and additionally for phase 2 at 12 months after the end of session 7, which included number of falls in the prior 6 months and FaB. If participants did not return the questionnaire by mail within 10 days, the evaluator called the individual to obtain data by phone. Three attempts were made to call by phone. Baseline and follow-up questionnaires included an information sheet explaining purpose, risks, benefits, and voluntary nature.

### Definitions for Phase 2 Evaluation

We defined four categories of rural versus urban based on the Rural–Urban Commuting Area (RUCA) codes [15]. RUCA codes combine US Census tract-based classification with work commuting information to characterize all US Census tracts on a gradient from rural to urban. A zip code approximation is available [16]. We defined the RUCA classification for workshop location and for participant residence from zip codes, and combined RUCA classifications to create four categories: urban (1.0, 1.1, 2.0, 2.1, 3.0, 4.1, 5.1, 7.1, 8.1, 10.1); large rural (4.0, 4.2, 5.0, 5.2, 6.0, 6.1); small rural (7.0, 7.2, 7.3, 7.4, 8.0, 8.2, 8.3, 8.4, 9.0, 9.1, 9.2); and isolated (10.0, 10.2, 10.3, 10.4, 10.5, 10.6).

We identified all leaders who had taught workshops according to their professional background. The primary professional backgrounds of leaders were PT, OT, or RN; health educator; and social worker; 1802 (74.6%) participants took workshops led by a leader from one of these backgrounds. The remainder of participants (623, 25.4%) took workshops led by leaders from other backgrounds: fitness leader, licensed practical nurse, speech therapist, PT assistant, executive director of a housing authority, medical technologist, registered dietician, lay volunteer, community partnership coordinator, Aging and Disability Resource Center director, owner of a home care agency, and owner of an assisted living facility. Leaders from these backgrounds were excluded from analysis of the association of type of leader with reduction in falls rates.

### Data Analysis

Mixed-effects Poisson regression models were used to evaluate the impact of Stepping On on the rate of falls. Models included intervention status (pre = baseline; post = 6- and 12-month follow-ups) as a fixed effect, and subject and the interaction between subject and intervention as random effects. Models were fit separately to all subjects and to prespecified subgroups based on leader type, subject, and site location.

Mixed-effects linear regression models were used to evaluate the impact of Stepping On on FaB scores. Models included intervention status (pre = baseline; post = 6- and 12-month follow-ups) as a fixed effect, and subject and the interaction between subject and intervention as random effects. Models were fit separately to all subjects and to prespecified subgroups based on leader type, subject, and site location. Analyses were performed using the lme 4 package [17] in R 2.13.1 [18].

## Results

### Phase 1 Implementation

**Fig. 2.**
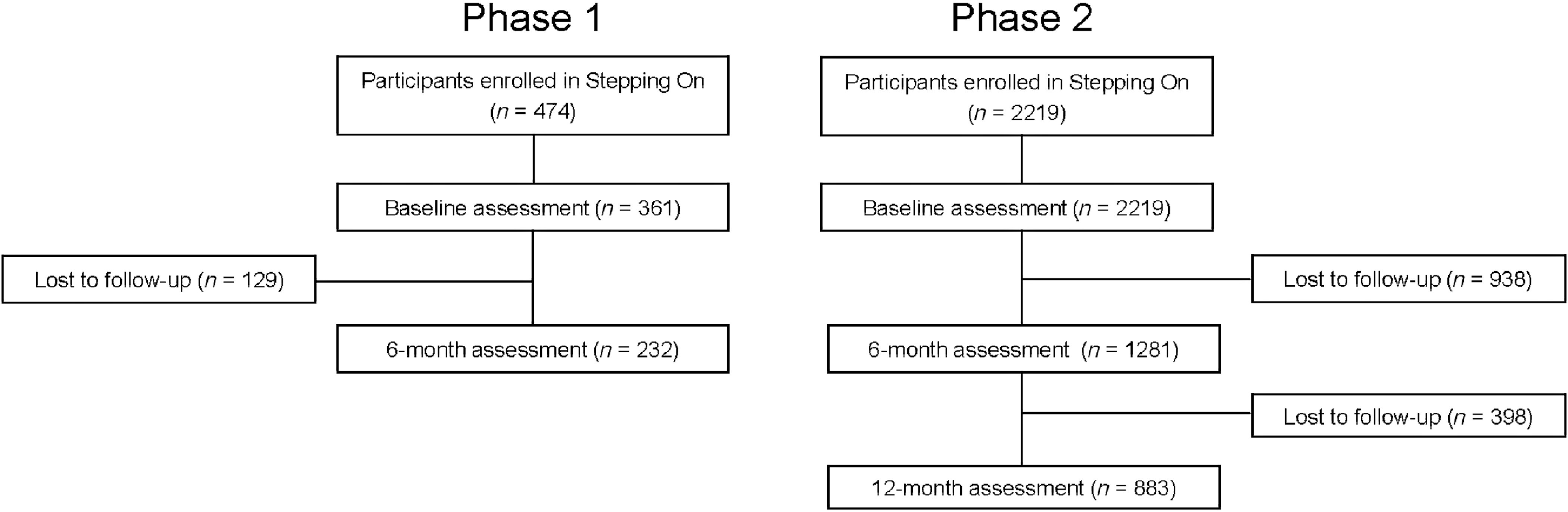
Flowchart describing enrolment and retention for phase 1 and 2 samples.

From July 2006 through January 2009, there were a total of 34 Stepping On workshops held in eight Wisconsin counties, enrolling 361 participants. Figure 2 shows enrollment and retention data for the phase 1 sample. Table 2 shows baseline characteristics of the sample; 96% of participants were over age 65, and 85% were over age 70; 85% were female. Table 3 shows the rate of falls at baseline, and the rate ratio for falls for the first 6 months after the intervention compared to 6 months before. The pre-intervention falls rate was 0.7 (95% CI 0.6–0.8) per 6 months. There was a non-significant 6% reduction in falls from the 6 months prior to the workshop to the 6 months after the workshop ended (RR = 0.94, 95% CI 0.74–1.20). There was an improvement of 0.14 points (95% CI 0.11–0.18) in FaB at 6 months associated with Stepping On participation, from a baseline score of 2.91 (95% CI 2.86–2.95).

**Table 2.** Characteristics of samples at baseline and follow-up

Phase 1 sample (September 2006–January 2009)			Phase 2 sample (January 2008–September 2011)			
	Baseline	6-month follow-up		Baseline	6-month follow-up	12-month follow-up
	(*N* = 361)	(*N* = 232)		(*N* = 2219)	(*N* = 1281)	(*N* = 883)
Age			Age (±SD)	78.1 (8.8)	78 (8.2)	77.6 (8.4)
<65	17 (5%)	9 (4%)	Missing, *n*	65	31	10
65–69	40 (11%)	26 (11%)				
70–74	64 (18%)	37 (16%)				
75–79	84 (23%)	52 (22%)				
80–84	74 (20%)	51 (22%)				
85–89	58 (16%)	40 (17%)				
90+	20 (6%)	14 (6%)				
Missing	4 (1%)	3 (1%)				
Sex			Sex			
Female	307 (85%)	203 (88%)	Female	1788 (81%)	1035 (81%)	710 (80%)
Male	53 (15%)	29 (12%)	Male	391 (18%)	229 (18%)	167 (19%)
Missing	1 (0%)	0 (0%)	Missing	40 (2%)	17 (1%)	6 (%)
Race/ethnicity			Race/ethnicity			
White	335 (93%)	215 (93%)	White	2105 (95%)	1229 (96%)	855 (97%)
Non-White	16 (4%)	13 (6%)	Non-White	58 (3%)	25 (2%)	19 (2%)
Missing	10 (3%)	4 (2%)	Missing	56 (3%)	27 (2%)	9 (1%)
Marital status			Marital status			
Never married	19 (5%)	11 (5%)	Never married	123 (6%)	75 (6%)	48 (5%)
Married	126 (35%)	75 (32%)	Married	843 (38%)	519 (41%)	379 (43%)
Divorced/separated	33 (9%)	18 (8%)	Divorced/separated	180 (8%)	80 (6%)	53 (6%)
Widowed	171 (47%)	120 (52%)	Widowed	1025 (46%)	586 (46%)	7 (1%)
Missing	12 (3%)	8 (3%)	Missing	48 (2%)	21 (2%)	7 (1%)
Location			Location			
City/suburb	284 (79%)	187 (81%)	City/suburb	1253 (56%)	725 (57%)	509 (58%)
Small town	52 (14%)	32 (14%)	Small town	620 (28%)	350 (27%)	220 (25%)
Rural/farm	22 (6%)	13 (6%)	Rural/farm	334 (15%)	201 (16%)	151 (17%)
Missing	3 (1%)	0 (0%)	Missing	12 (1%)	5 (0%)	3 (0%)
Use walking aid indoors			Use walking aid indoors			
No	290 (80%)	190 (82%)	No	1595 (72%)	951 (74%)	675 (76%)
Yes	54 (15%)	29 (12%)	Yes	548 (25%)	288 (22%)	180 (20%)
Missing	17 (5%)	13 (6%)	Missing	76 (3%)	42 (3%)	28 (3%)
Falls (±SD)	0.7 (1.2)	0.7 (1.2)	Falls (±SD)	1.2 (3.3)	1.1 (2.7)	1.1 (2.2)
Missing, *n*	30	18	Missing, *n*	201	114	74
Physician/emergency room visit for falls last 6 months			Physician/emergency room visit for falls last 6 months			
No	268 (74%)	167 (72%)	No	1813 (82%)	1061 (83%)	735 (83%)
Yes	66 (18%)	44 (19%)	Yes	303 (14%)	170 (13%)	116 (13%)
Missing	27 (7%)	21 (9%)	Missing	103 (5%)	50 (4%)	32 (4%)
Falls Behavioral Scale score (±SD)	2.9 (0.4)	3 (0.4)	Falls Behavioral Scale score (±SD)	2.9 (0.4)	2.9 (0.4)	2.9 (0.4)
Missing, *n*	5	4	Missing, *n*	131	77	34

**Table 3.** Change in the rate of falls and Falls Behavioral Scale scores associated with Stepping On workshop participation in phase 1 and phase 2

	Phase 1 sample			Phase 2 sample		
	Estimate	95% CI		Estimate	95% CI	
Falls						
Baseline falls rate (per 6 months)	0.7	0.6	0.8	1.2	1.0	1.3
Rate ratio (first 6 months)	0.94	0.74	1.20	0.62	0.57	0.68
Rate ratio (second 6 months)				0.72	0.65	0.80
Falls Behavioral Scale score						
Baseline	2.91	2.86	2.95	2.91	2.90	2.93
Change (at 6 months)	0.14	0.11	0.18	0.16	0.14	0.18
Change (at 12 months)				0.19	0.17	0.21

### Phase 2 Implementation

From January 2008 through June 2011, there were a total of 253 Stepping On workshops held in 43 of Wisconsin's 72 counties, enrolling 2219 participants. Three counties held 15 or more workshops, 6 held 10–14, 10 held 5–9, and 24 held 1–4. A total of 108 leaders held workshops. Figure 2 shows enrollment and retention data for the phase 2 sample. Baseline characteristics of participants are shown in Table 2. Participants’ mean age was 78, and 81% were female. Phase 1 and 2 samples were similar in marital status, race, and baseline FaB score. The phase 2 sample, compared to that of phase 1, had a slightly higher percentage using a walking aid indoors (25% versus 15%), and a higher baseline rate of falls per 6 months (1.2 versus 0.7). The samples were similar in the percentages seeking medical attention for a fall.

As shown in Table 3, Stepping On workshop participation was associated with a 38% reduction in falls from the 6 months prior to the workshop to the 6 months after the workshop ended (RR = 0.62, 95% CI 0.57–0.68). There was a 28% reduction in falls from the 6 months prior to the second 6 months after the workshop ended (RR = 0.72, 95% CI 0.65–0.80). There was a 0.16 improvement in FaB score from baseline to the first 6 months after the workshop ended (95% CI 0.14–0.18), and 0.19 score improvement from baseline to the second 6 months (95% CI 0.17–0.21).

### Effect of Leader Type on Workshop Participation and Outcomes

Table 4 shows similar rate ratios for falls with PT/OT/RN and health educator leader types. There was a greater reduction in falls with social workers as leaders compared to PT/OT/RN. There was no difference in change in FaB score according to leader type.

**Table 4.** Change in the rate of falls and Falls Behavioral Scale scores associated with type of Stepping On workshop leader, location of workshop, and location of workshop participants (phase 2 sample)

	Falls (per 6 months)					Falls Behavioral Scale score				
	*N* *	Rate ratio	95% CI		*p*-value	*N*	Change (at 6 months)	95% CI		*p*-value
Leader type										
PT/OT/RN	634	0.72	0.63	0.82		644	0.15	0.13	0.18	
Health educator	204	0.69	0.54	0.89	0.78	202	0.15	0.11	0.20	0.98
Social worker	188	0.50	0.38	0.65	0.02	190	0.17	0.12	0.22	0.43
Participant location										
Urban	800	0.56	0.49	0.63		805	0.16	0.14	0.18	
Large rural	195	0.76	0.60	0.96	0.02	198	0.12	0.07	0.17	0.19
Small rural	162	0.73	0.56	0.94	0.06	162	0.17	0.12	0.21	0.67
Isolated	224	0.71	0.55	0.91	0.09	224	0.20	0.15	0.24	0.09
Site location										
Urban	801	0.56	0.50	0.64		805	0.16	0.14	0.18	
Large rural	189	0.76	0.61	0.94	0.02	191	0.12	0.07	0.17	0.12
Small rural	225	0.71	0.55	0.93	0.11	225	0.17	0.13	0.22	0.52
Isolated	176	0.68	0.51	0.89	0.22	179	0.19	0.15	0.24	0.18

PT, physical therapist; OT, occupational therapist; RN, registered nurse.

* *N* = participants with follow-up at 6 or 12 months. Analysis of leader type included PTs, OTs, RNs, health educators, and social workers only. Participants in workshops led by other leader types were excluded from analysis. Analysis of participant location excluded participants with missing RUCA codes.

### Effect of Rurality on Workshop Participation and Outcomes

Table 4 shows locations of workshop participants’ residences, and locations of workshop sites for all attendees of Stepping On workshops. Over half of attendees were urban, with non-urban attendees being equally split among large and small rural, and isolated. Almost 60% of site locations were urban, with non-urban locations being equally split among large and small rural and isolated.

The effect of rural versus urban location on falls rate ratio (rate of falls 6 months after workshop divided by rate of falls 6 months before workshop) is shown in Table 4. Participants from urban residences had a greater reduction in falls compared to those from large or small rural or isolated residences, though the difference was only significant for the comparison with large rural residences. Results were similar by workshop location with participants in urban workshops having a larger reduction in falls, which was only significant in comparison with workshops at large rural sites. There were no significant differences in changes in FaB scores by participant location or site location.

We looked to see if type of leader, or participant or site location affected workshop attendance. Overall, 79% of participants attended five of seven sessions and were classified as completers. As shown in Table 5, the percentage of patients completing the workshop was significantly less for participants in small rural locations (69.8%) or attending small rural workshop sites (68.2%), compared to participants in urban locations (80.6%) or attending urban workshop sites (80.8%) (*p* = 0.0005 for all participant location comparisons versus urban location; *p* < 0.0001 for all workshop site comparisons versus urban workshop site).

**Table 5.** Likelihood of completing the workshop as a function of leader type and participant and workshop location (phase 2 sample)

	Completer**					
	*N* *	% completers	OR	95% CI		*p*-value
Leader type						
PT/OT/RN	1040	79.6	1.00			0.62
Health educator	302	77.5	0.88	0.65	1.21	
Social worker	277	77.6	0.89	0.65	1.23	
Participant location						0.0005
Urban	1264	80.6	1.00			
Large rural	270	81.9	1.08	0.78	1.54	
Small rural	308	69.8	0.56	0.42	0.73	
Isolated	291	79.7	0.95	0.69	1.31	
Site location						<0.0001
Urban	1282	80.8	1.00			
Large rural	289	81.3	1.03	0.75	1.44	
Small rural	305	68.2	0.51	0.39	0.67	
Isolated	283	79.8	0.94	0.69	1.31	

PT, physical therapist; OT, occupational therapist; RN, registered nurse.

* *N* = 2159 for analyses of completer status (60 participants had missing data on attendance). Analysis of leader type included PTs, OTs, RNs, health educators, and social workers only. Participants in workshops led by other leader types (*n* = 540) were excluded from analysis. Analysis of participant location excluded 26 participants with missing RUCA codes.

** Completer defined as attending five of seven sessions of Stepping On; 36 participants were listed as incomplete attendance and classified as non-completers.

## Discussion

This natural experiment afforded us the opportunity to see if an evidence-based program that was not effective when first implemented in a few counties in Wisconsin could become effective when disseminated statewide with attention to fidelity. Our results showed a significant reduction in rate of falls with the second, but not the first, phase of implementation of Stepping On in Wisconsin. The lack of effectiveness in phase 1 may be attributable to the multiple losses of fidelity when we originally adapted the program for dissemination in Wisconsin. The marked increase in effectiveness that we found in phase 2 may be due to the steps we took to ensure fidelity with dissemination. First, we identified key elements of Stepping On and developed a fidelity tool to track the delivery of key elements across all workshop sessions [11]. We then trained a new leader and monitored every session of the leaders’ first workshop for fidelity. Noting multiple areas where key elements were lost, we used a systems engineering approach (root cause analysis) to identify root causes in the training, the manual, the leader's background, the types of participants enrolled, and how they were enrolled [12]. We addressed these causes through multiple solutions: reworking the manual and training to emphasize key elements, ensuring leaders had a health professional background and previous experience working with behavior change groups, better preparing organizations to implement the workshop, changing how participants were recruited, and clarifying the criteria for the types of participants who would benefit from the workshop. We instituted fidelity monitoring and coaching for each new leader for an early session of their first workshop. We hypothesize that these changes to improve fidelity led to the program being effective in phase 2. The reduction in falls seen in phase 2 was similar to that seen in the original Stepping On randomized trial, suggesting that careful attention to fidelity can lead to the reproduction of original findings in the field, without the “voltage drop” that may often be seen [2]. Our results are further corroborated by a subsequent study of the effectiveness of Stepping On, which again found significant pre–post reductions in the rate of falls in association with workshop participation, as well as reduced fall risk behaviors (*p* < 0.001) and fewer medical record-verified emergency department visits for fall-related injuries (*p* < 0.05) [19].

There may be other reasons for our findings of improved reduction in falls with phase 2 versus phase 1. The second sample had a higher baseline rate of falls compared to the first sample, and it is possible that the sample in phase 1 was too high functioning to show a significant reduction in falls. The original Stepping On study also noted diminished program effectiveness with those at lower risk for falls.

We saw a significant improvement in FaB scores in both phases 1 and 2. Given this, we suspect that improvement in falls behavioral risk was not sufficient to account for the reduction in falls rate with Stepping On. The workshop aims to decrease behavioral risk, but also aims to improve balance and strength. Exercises are practiced in class; and building on adult learning and behavior change principles, participants are nudged successfully to practice the exercises daily at home and progress them in difficulty over time [20]. There is high-certainty evidence that balance and functional exercises can reduce the rate of falls, and moderate-certainty evidence that multiple types of exercise (typically balance, functional, and resistance exercises) can be effective [21]. In addition, participants cited changes to the way they walk and pay attention to the environment that may not be fully captured in FaB.

With phase 2, we saw no difference in rate ratio of falls in comparing program delivery by a health educator or a PT/OT/RN. The rate of falls was reduced more with workshops led by a social worker. It is unclear if this finding was due to chance or due to a difference in skills of social workers versus other health professionals. While traditionally social workers have not been considered as experts in falls prevention, many have expertise in promoting behavior change, which could enhance the effectiveness of program delivery. Overall, our findings confirm that workshops do not need to be led by a physical or occupational therapist or nurse to be successful. Individuals with other health-related backgrounds who have worked with older adults, and have experience with group facilitation using behavior change principles, can take the Stepping On leader training and succeed as leaders.

We had hypothesized that the program may not be as effective when delivered in rural compared to urban sites. In general, the reduction in falls rate tended to be higher for participants in urban locations, compared to large rural, small rural, or isolated locations; however, results were only significant for large rural versus urban. We hypothesized that this would, in part, be due to problems with attendance at rural locations, but attendance was only lower at small rural locations. Our finding of a higher reduction in falls in urban settings may be due to chance, but if true, it is unlikely that they are due to differences in attendance. We were unable to measure other factors, such as socioeconomic status or health literacy, that may have distinguished urban versus rural attendees and mediated their ability to benefit from the program.

This natural experiment represents an example of a successful application of dissemination and implementation science to improve impact with dissemination. In phase 1, we focused on maximizing adoption and sustainability. In phase 2, we used the REP framework to maximize fidelity and reach in both rural and urban areas without sacrificing adoptability or sustainability. Without the application of implementation science principles, we would not have been able to systematically identify key elements and identify and correct lapses in fidelity with broad dissemination. The REP framework has been used previously to guide adaptations of interventions for new settings or target groups, and/or develop strategies to overcome implementation barriers with spread [22–29]. However, there are little data on the utility of the REP framework to maintain fidelity and effectiveness with scale-up. Kind *et al.* [30] demonstrated that the use of a modified REP framework led to high-fidelity replication and effectiveness of a transitional care program in a different hospital setting than the original study, but they did not examine effectiveness with further scale-up. Jones *et al.* [31] described the use of the REP framework to field-test a community-based HIV/STD intervention for fidelity in three communities, but they did not report effectiveness with broad dissemination. Our results add to the literature by demonstrating the usefulness of the REP framework for ensuring fidelity and effectiveness with broad scale-up.

The Stepping On falls prevention program continues to be implemented, and its reach has expanded nationwide. It has now been implemented in 22 states besides Wisconsin, with over 35,000 total participants to date. The Wisconsin Institute for Healthy Aging (wihealthyaging.org) serves as the national purveyor. WIHA holds the nationwide license for Stepping On and can license other organizations to implement the program. The lead trainer at WIHA trains individuals at other organizations to first become leaders and then master trainers, who can train new leaders within their organization, following a train-the-trainer model. Master trainers observe all new leaders to ensure fidelity. As a condition of receiving the license, organizations annually report the number of leaders, workshops, and participants in the program.

This study has a number of strengths. We used the same methodology for data collection in phases 1 and 2. Between phases 1 and 2, we used rigorous methodology to identify key elements, identify lapses to fidelity, and revise the Stepping On program package, utilizing the REP dissemination and implementation framework. In addition, the reduction in falls rate seen in phase 2 was similar to that seen in the original Stepping On randomized trial, lending credence to the results.

There are a number of limitations to our work. First, falls were identified by retrospective self-report over a 6-month period. The gold standard for falls reporting is monthly calendar, but as funding was for program evaluation, we were not able to identify falls by the gold standard of monthly calendars [32,33]. Second, our findings are pre–post; we did not have a control group. Third, consistent with many program evaluations, in both samples, our response rate was low. It is possible that non-responders differed significantly from responders in ways that were not measured. Of note, the more recent waitlist cluster randomized trial of Stepping On by Ford *et al.* showed very similar results to those found here, adding strength to the validity of the results [19]. Fourth, the reach of Stepping On among communities of color was low. While the percentage of African-Americans and Native Americans in Wisconsin is relatively low compared to Caucasians, they were still underrepresented in our samples. More work needs to be done to determine if Stepping On can be adapted to reach communities of color in the United States. It should be noted that in Australia, Stepping On has reached indigenous populations.

In summary, this study of the two phases of implementation of Stepping On shows effectiveness in phase 2 that is temporally consistent with changes in program implementation to improve fidelity and effectiveness. Our findings suggest that with widespread dissemination of a complex behavior change intervention, effectiveness can be maintained if attention is paid to fidelity of key elements. Conversely, our study provides a cautionary note that dissemination of complex behavior change interventions without attention to fidelity may result in loss of program effectiveness. This study suggests an essential role for implementation science to ensure effectiveness as programs transition from research to practice.
